# An Unfortunate Young Man

**Published:** 1897-02

**Authors:** 


FOR THE BENEFIT of "an unfortunate young man," it is hoped that readers of the JOURNAL will contribute,* what they know of the management of such cases as his, that we may publish it.

It is true, no fixed rule of treatment will apply to all cases; and no one remedy will be efficient in all cases. The condition resulting from the abuse is to be treated on general principles, and it will vary in degree and intensity. Tonics are indicated, usually. "Iron, steel and bark," were the old time stand-bys; cold baths to the spine, in the way of a douche; electricity, gymnastics, etc., have all been used, with more or less benefit; but something else is needed. It may be that the condition calls for nerve-sedation, general or special; or it may be that the reverse is indicated.

---

*See correspondence.

In all cases, the first requisite is to *quit the practice.* True, it may be hard to do, and the unfortunate may have already passed the stage when any effort of the will will avail; yet without a cessation of the practice no relief is to be expected. The unfortunate should brace himself to a supreme effort, and let nothing induce him to break his resolve to abandon the destructive habit; for unless at once and forever abandoned, it is only matter of very short time when he will become a most loathsome wreck, mentally and physically. After breaking the habit, scrupulous cleanliness, frequent general baths, and especially cold douches to the parts and to the spine are to be recommended; to abstain from all thoughts that have a bearing in that direction, and from all alcoholic or other stimulants; to sleep under light covering, and on a hard mattrass, etc. This, with nutrition diet, and the internal use of strychnia, is about all we can suggest in a general way. If there be frequent priapism, the use of the bromides, with camphor or hyoscyamus would be beneficial; a cold steel sound in the urethra has been advised.

It has been recommended to cauterize the glans penis; to create a sore, which would make the act painful, and thus restrain the party; but, if abstinence be not the effect of will power, we apprehend that the sore would not be of much service. Matrimony is, unquestionably, the remedy, in a large number of cases; but, in this young man's case, it will be observed, the organs are undeveloped, and matrimony would be a failure, essentially.

The above has been written out of a sincere desire to help the young man who so pathetically appeals to the JOURNAL for help, and to create an interest in his case in the minds of our readers, in the hope that some among them may have had experience in the treatment of cases like his, and can offer him some hope. The unfortunate is totally unknown to us; and while we are in the habit of paying no attention to anonymous letters—yet his case appeals so strongly to our sympathy, that we thus publish it and ask for suggestions for his benefit. Our best advice is to put himself in the hands of some competent and conscientious practitioner, for treatment; say, at some private sanitarium or "Home" or "Retreat."

Should all other means fail of cure, and, especially, as the organs are undeveloped, and the essential purposes of matrimony are out of the question, we unhesitatingly advise the extirpation of the glands and their appendages,—a clean emasculation.—It may be the only thing that can save him from a fate a thousand times worse than death.

